# Negative consequences of glacial turbidity for the survival of freshwater planktonic heterotrophic flagellates

**DOI:** 10.1038/srep04113

**Published:** 2014-02-17

**Authors:** Ruben Sommaruga, Georg Kandolf

**Affiliations:** 1 University of Innsbruck, Institute of Ecology, Alpine Freshwater Ecology Division, Lake and Glacier Research Group. Technikerstr. 25, 6020 Innsbruck, Austria

## Abstract

Heterotrophic (phagotrophic) flagellates are key components of planktonic food webs in freshwater and marine ecosystems because they are the main consumers of bacteria. Although they are ubiquitous in aquatic ecosystems, they were numerically undetectable in turbid glacier-fed lakes. Here we show that glacial particles had negative effects on the survival and growth of heterotrophic flagellates. The effect of glacial particles was concentration-dependent and was caused by their interference with bacterial uptake rather than by physical damage. These results are the first to reveal why establishment of heterotrophic flagellates populations is hindered in very turbid glacial lakes. Because glaciers are vanishing around the world, recently formed turbid meltwater lakes represent an excellent opportunity to understand the environmental conditions that probably shaped the establishment of lake communities at the end of the last glaciation.

One of the most prominent signs of climate change is the worldwide retreat of glaciers^1^,^2^. One consequence of vanishing glaciers is the on-going formation of many new proglacial lakes around the world^3^. Proglacial lakes are characterized by high water turbidity produced by minerogenic particles, so-called glacial ‘flour'. Glacial particles originate from the crushing and abrasion of bedrock by the glacier and, due to their small size, they can be suspended in the water column for long periods^4^. The recent climatic warming and associated melting of glaciers can further increase the turbidity of lakes connected to glaciers and extend the periods of high particle loads during the ice-free season. On the other hand, when lakes become disconnected from glacial influence, they turn clear.

Little is known about the effect of glacial ‘flour' on lake biota, but filter-feeding species such as the water flea *Daphnia* are usually absent in oligotrophic lakes influenced by high glacial ‘flour' concentrations^5^ and the vertical distribution and community composition of phytoplankton and zooplankton is affected by the discharge of turbid glacial meltwaters into clear lakes^6^. Paleoecological studies have also documented extensive collapse of lake ecosystems during the last glacial maximum^4^, and the discharge of glacial turbid water into coastal waters has been putatively associated with increased zooplankton mortality^7^. The presence of large amounts of suspended minerogenic particles probably has negative consequences for other taxa of planktonic organisms such as heterotrophic nanoflagellates (HNF). Together with viruses, this group of organisms, usually described as bacterivores or phagotrophic flagellates, is one of the main agents of mortality for prokaryotes. Heterotrophic nanoflagellates are ubiquitous in marine ecosystems, lakes, rivers, and estuaries^9^ where they typically reach abundances between 100 and 10,000 cells ml^−1^. Because they channel organic matter to larger planktonic organisms^8^, and they are so abundant, they play a pivotal role in aquatic ecosystems.

In this work, we investigate whether suspended glacial particles have a negative effect on HNF in order to explain their apparent absence in turbid glacial alpine lakes. We compare the effect of glacial ‘flour' on a natural flagellate community, as well as on experimental model flagellate species having different nutritional strategies. Further, we test the potential of glacial particles to interfere with HNF feeding. Since we expected the glacial particles to have different characteristics compared to those found in other turbid systems (e.g., shallow turbid lakes where turbidity is increased through sediment resuspension)^10^, we also investigate their organic carbon coating and particle size distribution. We hypothesized that glacial particles negatively affect the growth/survival of strict phagotrophic nanoflagellates due to their interference with bacteria, the dominant prey. We further anticipated that physical damage by ingestion of sub-micron minerogenic particles increases flagellate mortality and that these two mechanisms operate at different time scales (e.g., shorter in the case of physical damage). Therefore, we expected that addition of glacial ‘flour' to a natural HNF assemblage from a clear lake, at concentrations found in turbid glacial lakes, will cause a significant decrease in flagellate survival. Finally, we expected that mixotrophic flagellate species might cope with the interference of glacial particles because they are able to shift their nutritional strategy to a photosynthetic one.

## Results

### Characterization of glacial particles

**Figure 1:**
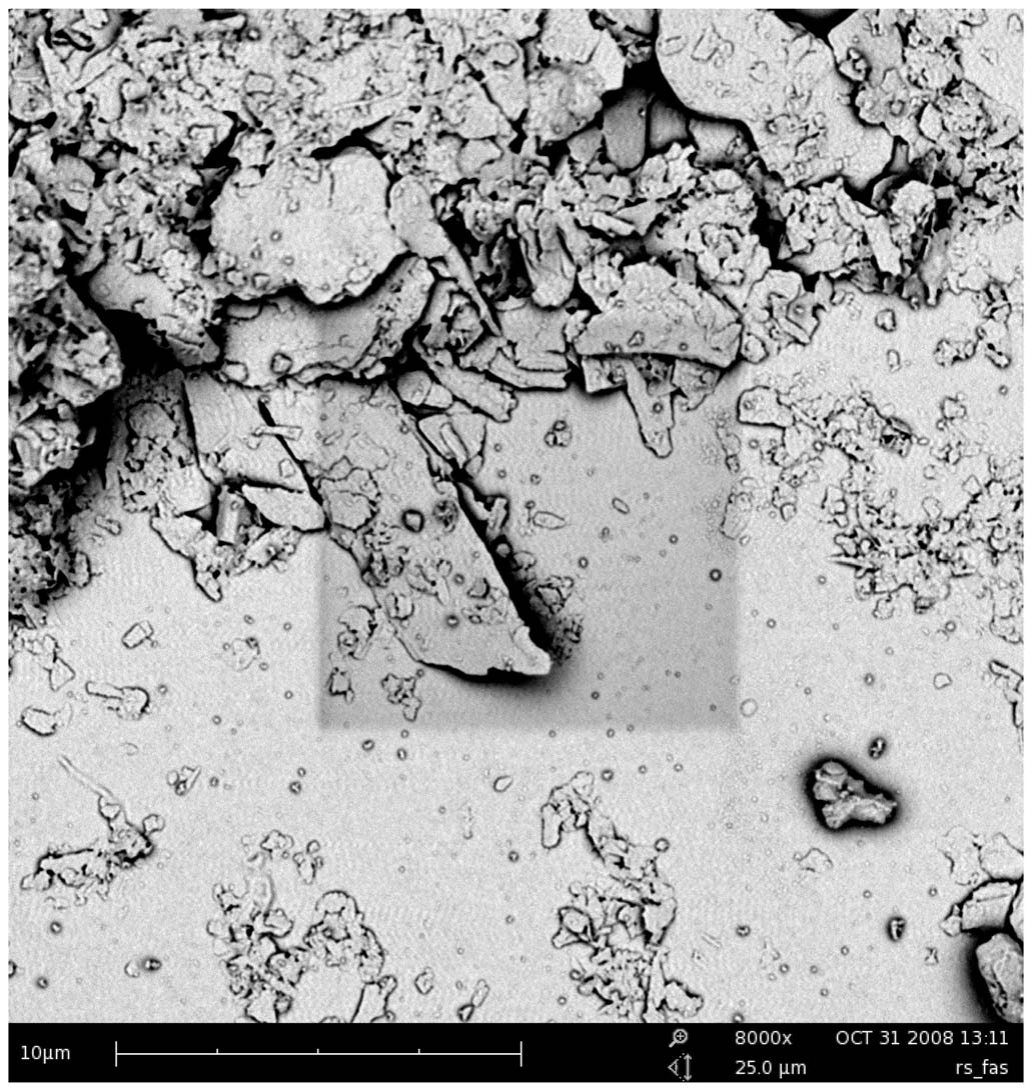
Scanning electron micrograph (SEM) of glacial ‘flour' from Lake Faselfad 1 showing particles of *ca.* 0.2 to 3 μm in diameter.

Scanning electron microscopy (SEM) revealed that glacial particles were characterized by sharp edges (Fig. 1) and, in the youngest proglacial turbid lake (FAS1), were dominated by muscovite/illite (57%) followed by chlorite (31%) and quartz (12%). In older lakes in the deglaciation chronosequence, such as in Lake FAS3, particles were similarly dominated by muscovite/illite (55%), but also calcite (22%) was present. These clays are grey-white to silvery-white, which is also the typical hue of the water in new proglacial lakes (see Supplementary Fig. S1 online). Under the epifluorescence microscope, the particles fluoresced yellow (Supplementary Fig. S2 online). The average relative content of carbon and nitrogen in the glacial ‘flour' was 0.47% and 0.060%, respectively, which is consistent with the low weight-loss-on-ignition value (0.81%). Most of the particles (98%) - analyzed using a Coulter Counter - were in the size range 0.7–4 μm and at 14 nephelometric turbidity units (NTU), their abundance represented 2.83 × 10^6^ ml^−1^ (Supplementary Fig. S3 online). Laser diffractometry revealed that particles <2 μm represented a low percentage of the scattered volume (<3%), whereas 50% of the scattered volume was produced by particles <25 μm (Supplementary Fig. S3).

### Abundance of bacteria and HNF in clear and turbid FAS lakes

The abundance of HNF in Lake FAS4, a system that is disconnected from the glacier and became clear (Supplementary Fig. S1, S2), ranged from 0.85 to 2.57 × 10^3^ cells ml^−1^ and was higher in deep water layers than at the surface (Supplementary Table S1). In the turbid lakes, FAS1 and FAS3, HNF were not observed despite our having filtered large volumes of water. The addition of a HNF culture to those samples resulted in a positive detection. In contrast, the abundance of autotrophic nanoflagellates was higher in Lake FAS3, followed by FAS4 and FAS1 (Supplementary Table S1 online). Bacterial abundance was lowest in FAS1 (1.93 × 10^5^ cells ml^−1^) and highest in FAS4 with the maximum (8.67 × 10^5^ cells ml^−1^) observed in deep water layers (Supplementary Table S1).

### Effect of glacial ‘flour' on a natural flagellate community

**Figure 2:**
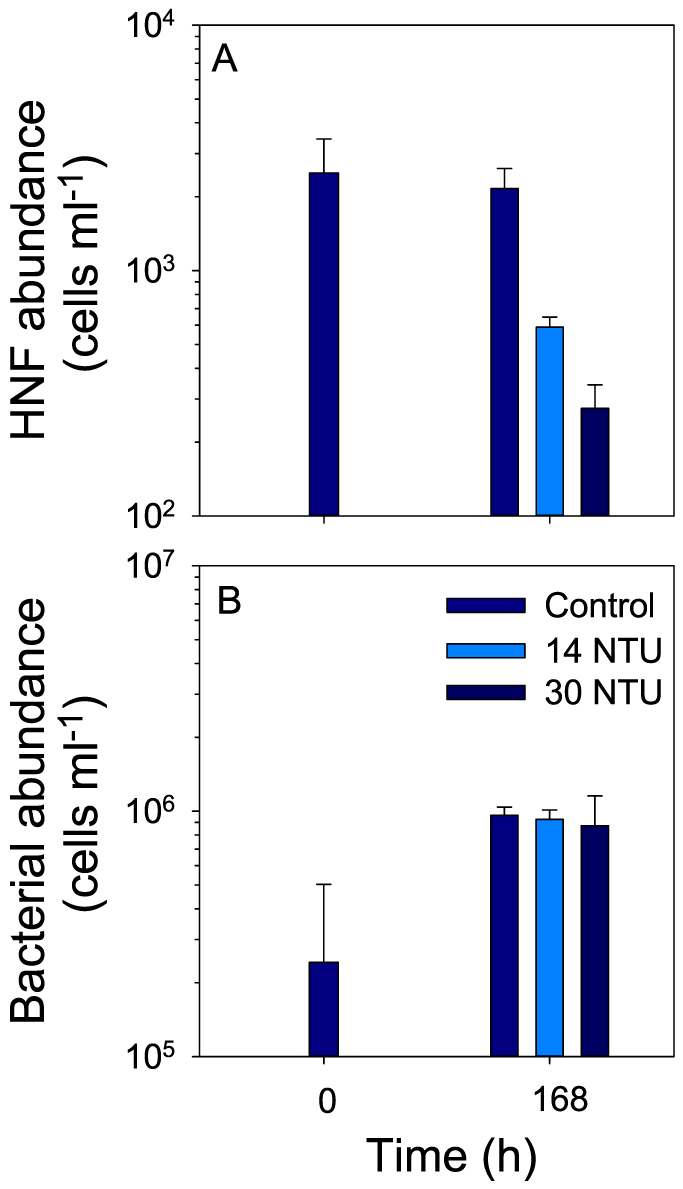
Effect of glacial ‘flour' on the abundance of a natural heterotrophic nanoflagellates (HNF) community (A) and on bacteria (B) after 168 h. Error bars represent ± 1SD.

To quantify the effect of glacial particles on a natural flagellate community, two different turbidity concentrations were tested on a sample collected from Lake FAS4. There was no significant difference in HNF abundance in the control, from the start to the end of the experiment (Holm-Sidak post-hoc test, p = 0.445) (Fig. 2a). However, in both treatments, the number of HNF dropped significantly after one week in comparison to the control (ANOVA F_(3,8)_ = 13.62, p = 0.002, all pairwise multiple comparisons with Holm-Sidak significant to p < 0.01). The decrease in HNF cells was most evident in the 30 NTU treatment. Bacterial abundance in the control and in the 14 NTU and 30 NTU treatments increased significantly from the beginning to the end of the experiment (F_(3,8)_ = 8.69, p = 0.007). However, after 1 week, bacterial abundance was not significantly different between the control and the two treatments (Holm-Sidak post-hoc test). As a consequence of the change in bacterial abundance, the bacteria (1–3 μm):particle ratio was higher at the end of the experiment in both treatments (Supplementary Fig. S4 online).

### Effect of glacial ‘flour' on experimental model flagellate species

**Figure 3:**
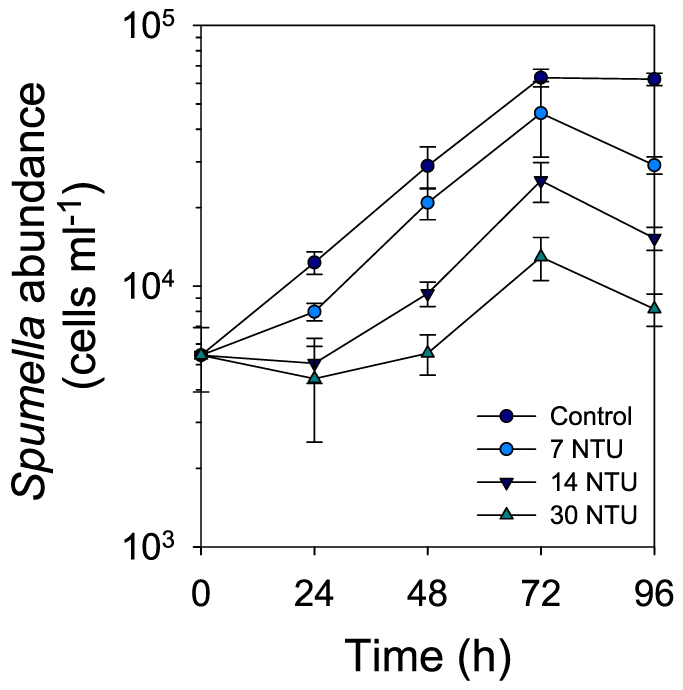
Growth of *Spumella* sp. in the control and in the presence of glacial ‘flour' at 7, 14, and 30 NTU. Error bars represent ± 1SD.

**Figure 4:**
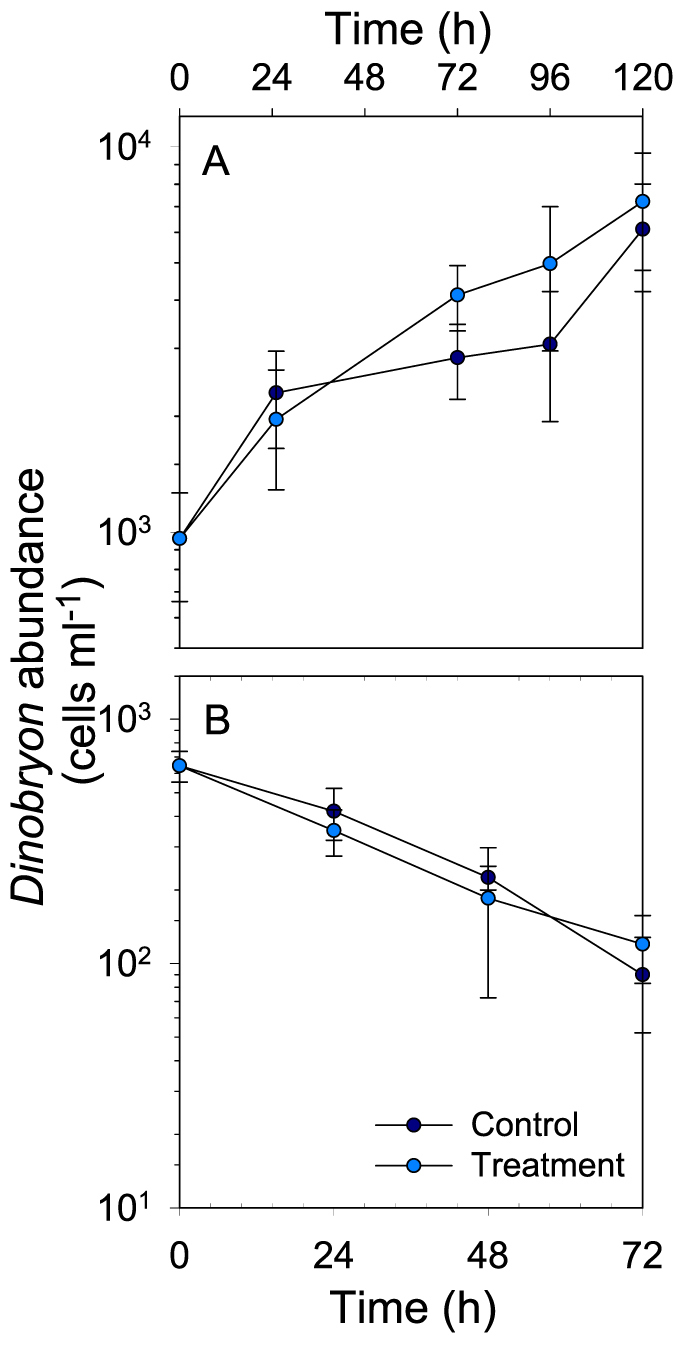
Changes over time in the abundance of *Dinobryon divergens* when grown in the presence of light (A) or in darkness (B), and in the absence (control) and in the presence of glacial ‘flour' (treatment). Error bars represent ± 1SD.

The abundance of the strict heterotrophic *Spumella* sp. was significantly different among treatments and control (F_(4,3)_ = 5.99, p = 0.01, Fig. 3). The negative effect of glacial ‘flour' on growth was clearly concentration-dependent (30 NTU > 14 NTU > 7 NTU), though the difference between the control and the 7 NTU treatment was not significant (Holm-Sidak post-hoc test p = 0.096). In contrast, in experiments with the mixotrophic species *Dinobryon divergens* conducted under dark and light conditions, no significant difference was found between its abundance in the control and the treatment (experiment with PAR: F_(4,1)_ = 3.60, p = 0.131, Fig. 4a; experiment in darkness: F_(3,1)_ = 0.83, p = 0.430, Fig. 4b). Abundance in the control and treatment decreased continuously during the experiment done in the dark suggesting that phagotrophy was not enough to sustain the population or that the switch from light to darkness was too abrupt for adaptation to the new conditions to occur.

### Interference of glacial ‘flour' with grazing

**Figure 5:**
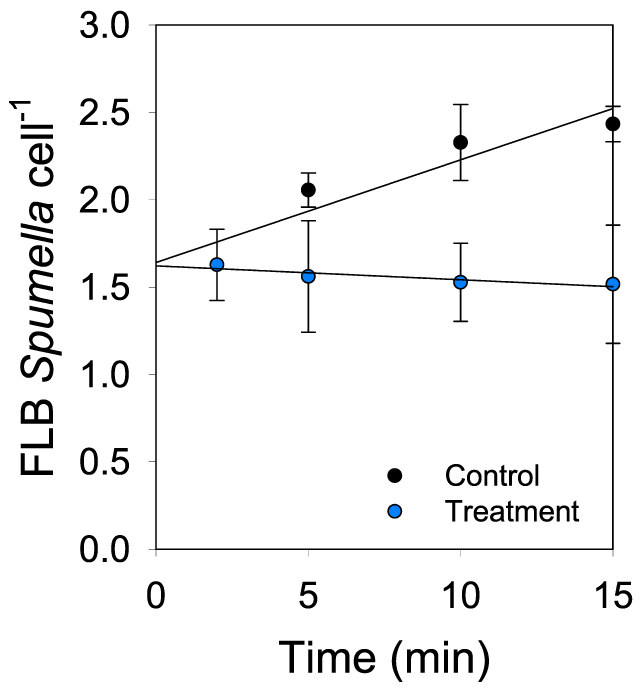
Changes over time in the average number of fluorescently labeled bacteria (FLB) ingested per *Spumella* cell in the absence (control) and in the presence of glacial ‘flour' (treatment) corresponding to 14 NTU. Error bars represent ± 1SD.

A short-term uptake experiment using fluorescently labelled bacteria (FLB) as surrogates indicated that the grazing rate of *Spumella* sp. was significantly higher in the control (Fig. 5) than in the treatment (comparison of the slopes of the two regressions t = 2.945, p < 0.05). Thus, whereas the uptake rate (UR) in the control was 5.16 FLB cell^−1^ h^−1^ corresponding to a clearance rate (CR) of 0.023 nL cell^−1^ h^−1^, in the treatment, the UR was 1.14 FLB cell^−1^ h^−1^ and the CR was 0.0051 nL cell^−1^ h^−1^.

## Discussion

Our results clearly demonstrate that the presence of glacial particles had a negative effect on the survival of a natural HNF community (Fig. 2a). Furthermore, the negative effect of the glacial ‘flour' on the HNF community was concentration-dependent, i.e., the highest mortality was observed in the treatment with the highest turbidity. Overall, these results suggest that members of a natural HNF community from clear water lakes, such as that from Lake FAS4, will not survive under conditions of high mineral particle concentration and that glacial ‘flour' hinders the establishment of HNF populations in glacial turbid lakes. Although we did not identify which HNF taxa were present in this natural community and, consequently, whether all species were affected in the same way, the large decrease in abundance suggests that the negative effect was widespread. There are also other potential factors which could decrease the chances of colonization of (pro)glacial lakes by HNF. One could be food limitation. This apparently occurs when bacterial numbers are <10^6^ cells ml^−1^ (Ref. 9). Thus, bacterial abundances of 10^5^ ml^−1^ typically found in alpine lakes such as the FAS suggest that HNF could be at times food-limited. However, in alpine clear water lakes, HNF populations exist indicating that bacterial abundance is not relevant for establishing a community. In fact, the clear water Lake FAS4 had a bacterial abundance similar to that of Lake FAS3, but HNF were present. Nevertheless, the presence of high numbers of particles implies that search for and handling of a bacterium by a HNF in a diluted food medium takes more time and uses more energy^11^.

The results from the experiments with different flagellate species provided evidence for the mechanism behind the negative effect of glacial particles. The experiment with *Spumella* sp. (Fig. 3) clearly showed that growth was negatively affected by glacial ‘flour' in a concentration-dependent manner and that HNF growth reduction is caused by a reduced grazing efficiency (Fig. 5). In the absence of glacial ‘flour', the uptake and clearance rates were comparable to those reported for *Spumella* species with the same cell size range as the strain used in our experiments^12^. In our experiments with *Spumella* sp., the bacteria:particle ratio was much higher than that found in proglacial lakes such as in Lake FAS1 (Supplementary Fig. S4). However, despite the potential higher contact rate between flagellates and bacteria, a negative effect was evident. A much stronger negative effect can be expected when the bacteria:particles ratio is low as found in lakes FAS1 or FAS3. This is feasible because the results from the size distribution analysis indicated that a significant fraction of the glacial particles (Supplementary Fig. S3) lies within the size range of bacteria preferentially consumed (1–3 μm) by phagotrophic flagellates^13^. In this respect, our results resemble those obtained for *Daphnia*, for which survivorship in the presence of glacial ‘flour' was dependent on the algal biomass available^14^.

Another potential mechanism for the reduced HNF survival in the presence of glacial ‘flour' can be physical damage. In fact, glacially-derived suspended particles had sharp edges (Fig. 1) and were dominated by clays (<4 μm). This is consistent with results for other lakes where glacial particles of 0.1 μm diameter are dominant^15^. As we hypothesized, these submicron particles can be damaging if ingested together with bacteria. The fact that we did not observe a significant reduction in flagellate abundance immediately after addition of the glacial ‘flour' suggests that the negative effect observed in *Spumella* sp. was not caused by physical damage, as this is expected to occur shortly after its addition.

Planktonic flagellates are often associated with (organic) particles and this contact can be either permanent or temporary^9^. Attachment to particles or biofilms can significantly increase feeding efficiency because the performance of the flagellum-induced feeding currents is enhanced^16^. Some species of HNF (e.g., *Bodo* sp.) have a striking ability to feed on particle-associated bacteria^17^ and, when coated with an organic layer, suspended particles are usually hot-spots of microbial activity^18^,^19^. However, glacial particles had very low organic content and were never seen colonized by bacteria. This is consistent with findings from other glacial lakes, as well as subglacial sediments and waters^20^,^21^,^22^. In addition, the weight-loss-on-ignition indicated low organic coating when compared with values for sediments (~1–35% of dry weight) from proglacial and non-glacial lakes^23^.

In contrast to *Spumella* sp., *D. divergens* is a mixotrophic species insensitive to glacial ‘flour' in the presence of light (Fig. 4a). The lack of a negative effect of glacial ‘flour' on this mixotrophic species suggest that flagellates with this nutritional mode could be favored in turbid glacial lakes. Although this hypothesis needs to be tested, we observed that although HNF where not found in lakes FAS1 and FAS3, phototrophic flagellates - probably including mixotrophic ones - were present in those lakes at even higher abundance than in clear Lake FAS4.

As a result of the current rapid glacier retreat due to climate change, many new proglacial lakes are emerging^3^. These newly formed water bodies provide unique harsh conditions that hinder establishment of key organismal groups such as HNF and cladocerans. Because HNF efficiently channel organic matter to higher trophic levels^8^, their absence in glacial turbid meltwater lakes, together with that of keynote planktonic species such as *Daphnia,* suggests that their food-web is partially truncated. This community structure places copepods such as *Cyclops abyssorum* as probably major consumers of nanoplankton in lakes such as the Faselfad ones. We argue that this type of lake has a special microbial food-web structure that probably resembles that present during the period of lake formation at the end of the last glacial period. The absence of HNF, and the probable interference of glacial particles with viruses, should have important consequences for the function and structure of prokaryotic communities in those lakes, an issue that we are currently studying.

## Methods

### Origin of the glacial ‘flour'

Surface water samples to obtain glacial particles were collected with a modified Schindler-Patalas sampler from the turbid lakes FAS1 and FAS3. These lakes are located in the Austrian Alps (47°04′25–37″N 10°13′21–28″E) and together with four other systems in this area represent a turbidity gradient resulting from a deglaciation chronosequence (Supplementary Fig. S1). Water samples were placed into 10 L carboys for 6 months in the dark at 4°C to let the particles settle. Then, most of the water was extracted by suction and the remaining water, including the sediment, was dried at 45°C for 5 days. In addition, sediment samples were taken from FAS1 with a plastic core and the upper 1 mm of the sediment was removed and dried in the same manner. The dried material was then ground and screened through a metal mesh sieve (mesh size 63 μm), although no sediment material was retained. The glacial particles used for the experiments and further characterization (see Supplementary Methods) are, therefore, the sum of the settled particles plus those in the upper sediment.

### Turbidity

This parameter was measured by nephelometry using a handheld turbidimeter (WTW Turb 430 T) that uses ‘white light' emitted by a tungsten lamp. The method follows the protocol recommended by the US EPA (#180.1). A three-point calibration was done with standards of 0.02, 10, and 1000 NTU. For each sample, three measurements were made and the mean value was calculated.

### Abundance of bacteria and flagellates in clear and turbid FAS lakes

To obtain reference values on the bacterial and flagellate abundance in the Faselfad lakes, water samples were collected from FAS1 (1 m depth), FAS3 (1 m, 6 m, 10 m, 15 m depth), and clear FAS4 (1 m, 6 m, 10 m, 13 m depth) during a summer field campaign. Water samples were collected using the same type of sampler, fixed in the field with formalin (2% final concentration), and transported by helicopter to the laboratory in Innsbruck in containers that were kept cool and dark.

For the analysis of bacterial abundance, subsamples of 5 to 15 mL were stained for 5 min with the fluorochrome 4′6′-diamidino-2-phenylindole (DAPI) (10 μg mL^−1^) and then concentrated onto black polycarbonate membrane filters (pore size 0.2 μm). At least 400 cells were counted under an epifluorescence microscope (Zeiss Axiophot 2) at a magnification of 1250×. For counting flagellates, the preserved subsamples (20–80 mL) were stained and counted as for bacteria, but filtered onto black polycarbonate membrane filters with a pore size of 0.8 μm. When no HNF were detected, more water was filtered. In additional tests, a small volume of a HNF culture was added to the sample to make sure that they could be detected in the presence of particles. To distinguish between heterotrophic and autotrophic flagellates, individuals were checked for the presence of chlorophyll-*a* in autofluorescing plastids by using the filter set with blue excitation (excitation 450–490 nm, emission filter LP 520 nm).

### Effect of glacial ‘flour' on a natural flagellate community from a clear high mountain lake

On 5 July 2011, a 5 L water sample was collected from Lake FAS4 at 13 m depth (typically the depth at which there was maximum HNF abundance) and transported to the laboratory in Innsbruck by helicopter in a plastic carboy that was kept in the dark and close to the *in situ* temperature. This sample was used to test the effect of two different particle concentrations (14 NTU; 57 mg L^−1^ and 30 NTU: 113 mg L^−1^) on the natural assemblage of flagellates. To exclude large organisms, the sample was screened through a zooplankton net (45 μm mesh size) and distributed into 100 mL glass bottles that were daily shaken to avoid sedimentation. The experiment was conducted in the darkness and at 15°C using three replicates for the treatments and control (i.e., no particles added), respectively. Bottles were briefly opened every day to allow exchange of air. The abundance of bacteria and HNF were determined at the start of the experiment and after 1 week as described above.

### Test organisms and culture conditions

Most experiments were done with the HNF species *Spumella* sp. strain JBM10 (diameter 4–7 μm), which is a common cryptomonad genus in freshwater communities^24^. This species can be classified as an interception feeder that grazes on small- to medium-sized bacteria^17^. For comparison with another phagotrophic, but at the same time phototrophic (i.e., mixotrophic) species, we included the chrysophycean *Dinobryon divergens* (length 12–15 μm), which is a flagellate that often forms colonies and is commonly found in freshwaters and also in high mountain lakes. *Spumella* sp. and *D*. *divergens* were provided courtesy of Jens Boenigk. *Spumella* sp. was grown in inorganic basal medium (IBM) supplemented with 2.5 mg L^−1^ of glucose and 40 mg L^−1^ of peptone. *D. divergens* was cultured in WCg-medium^25^. Cultures were kept in an environmental chamber with a light:dark photoperiod of 8:16 h in transparent 50 mL polystyrene tubes at 23°C and were transferred to fresh medium every 2–3 weeks.

### Effect of glacial ‘flour' on flagellate growth

Two days prior to the experiments, 1 ml of culture and 9 mL of fresh medium were transferred into a 15 mL tube. At the start of the experiment, 100 μL of culture and 900 μL of fresh medium were transferred into 2 ml Eppendorf vials. The experiment was run using five replicates for the treatment and control (i.e., no glacial ‘flour' added), respectively. Glacial particles were added to the treatment at a concentration of 57 mg dry weight L^−1^ (14 NTU) in the experiment with *D. divergens* and at 7, 14, 30 NTU in the case of *Spumella* sp. These turbidity values are moderate and can be one order of magnitude higher in other turbid glacial meltwater lakes^26^. The experiments were conducted at 15°C and in darkness, except for *D. divergens* for which additionally the effect was tested in the presence of light, with a light:dark photoperiod 8:16 h at 23°C. The pH-value in the treatment was checked after addition of the glacial ‘flour'. This measurement revealed that addition of glacial ‘flour' did not extensively alter the pH (control 7.09, treatment 7.05). To avoid sedimentation of the particles, all vials (i.e., also the controls) were placed in a rotator (VWR Tube Rotator) that has a fixed speed of 18 rpm. Rotation mode was adjusted to 15 min (on) and 45 min (off) cycles to avoid potential shear stress on the flagellates. Changes in flagellate abundance over time were assessed by counting cells in drops of 0.5–2 μL under an Olympus BX 50 (brightfield, digital interference contrast) microscope at 40–200× magnification^27^. At least 200 cells were counted at time intervals of 24 h. Bacterial numbers were counted at the same time intervals as described above.

### Short-term uptake experiment with fluorescently labeled bacteria

The grazing rate of *Spumella* sp. was determined by using fluorescently labeled bacteria (FLB) as surrogates^28^. Bacteria were stained with the yellow-green fluorescing dye, 5-(4,6-dichlorotriazin-2-yl) aminofluorescein (DTAF). The bacteria used to prepare the FLB were those growing together with *Spumella* sp. and are a mixed culture. To remove the flagellates, the medium was filtered through a 2 μm pore size polycarbonate filter. To estimate the concentration of FLB in the suspension, cell abundance in a small sample (0.5 ml) was counted under the epifluorescence microscope at 1250× magnification. For the experiment, the *Spumella* sp. culture (10 ml) was first inoculated into a 500 ml glass bottle holding 390 ml IBM medium and, after 5 days, the dense flagellate culture was transferred to 50 ml tubes with three replicates for the treatment and control, respectively. To estimate the required volume of FLB to add (10–15% of the total bacteria abundance, ref. 28), a sample (2 ml) was fixed and stained and bacteria enumerated as described above. Control and treatment tubes were then supplemented with FLB and FLB plus glacial particles (57 mg L^−1^), respectively. The experiment was run in the darkness at 16°C. Subsamples of 5 ml were taken at time 0 and after 2, 5, 10, and 15 min. The subsamples were immediately fixed with 0.25 μL alkaline Lugol's solution (0.5%) and 0.25 mL buffered formalin (40%), and stored in the dark at 4°C until processed within 24 h. Subsamples were then stained with DAPI and concentrated onto black polycarbonate filters (0.8 μm pore-size). Flagellates were examined under an epifluorescence microscope at a magnification of 1250×. The number of ingested FLB per individual was assessed by switching between the filter-set for UV (DAPI) and blue light excitation (DTAF). At least 60 individuals were checked on each filter. The uptake rate (UR) in FLB cell^−1^ h^−1^ was obtained by extrapolation of the regression slope between FLB cell^−1^ against time at 60 min. The clearance rate (CR) in nL cell^−1^ h^−1^ was calculated by dividing the UR by the number of FLB used in the experiment^28^.

### Statistics

Results of the experiments on the effect of glacial ‘flour' on the short-term uptake experiment were analyzed by comparing the slopes of linear regressions between the control and treatments with a student t-test^29^. The results from the experiments to test the effect of different particle concentrations on *Spumella* sp. and on the natural community experiment were analyzed using a one-way ANOVA (repeated measurements when different times were included) with Holm-Sidak post-hoc test. The analyses were done using the Systat software SigmaStat 3.5 and the StatSoft software STATISTICA 9.

## Author Contributions

R.S. designed the experiments and G.K. performed the experiments and field sampling. Both authors wrote the manuscript and prepared the figures.

## Supplementary Material

Supplementary Information Supplementary Information for Negative consequences of glacial turbidity for the survival of freshwater planktonic heterotrophic flagellates by Sommaruga and Kandolf

## References

[b1] Kääb A. *et al.* Contrasting patterns of early twenty-first-century glacier mass change in the Himalayas. Nature 488, 495–498 (2012). 22914167 10.1038/nature11324

[b2] Zemp M. Alpine glaciers to disappear within decades? Geophys. Res. Let. 33, LXXXXX, 10.1029/2006GL026319 (2006).

[b3] Frey H. *et al.* A multi-level strategy for anticipating future glacier lake formation and associated hazard potentials. Nat. Hazards Earth Syst. Sci. 10, 339–352 (2010).

[b4] Karabanov E. *et al.* Ecological collapse of Lake Baikal and Lake Hovsgol ecosystems during the Last Glacial and consequences for aquatic species diversity. Palaeogeogr. Palaeoclimatol. Palaeoecol. 209, 227–243 (2004).

[b5] Koenings J. P., Burkett R. D. & Edmundson J. M. The exclusion of limnetic cladocera from turbid glacier-meltwater lakes. Ecology 71, 57–67 (1990).

[b6] Hylander S. *et al.* Climate-induced input of turbid glacial meltwater affects vertical distribution and community composition of phyto- and zooplankton. J. Plankton Res. 33, 1239–1248 (2011).

[b7] Węsławski J. M. & Legez·yńska J. Glaciers caused zooplankton mortality? J. Plankton Res. 20, 1233–1240 (1998).

[b8] Azam F. *et al.* The ecological role of water-column microbes in the sea. Mar. Ecol. Prog. Ser. 10, 257–63 (1983).

[b9] Boenigk J. & Arndt H. Bacterivory by heterotrophic flagellates: community structure and feeding strategies. Antonie von Leeuwenhoek, 81, 465–480 (2002). 10.1023/a:1020509305868 12448743

[b10] Scheffer M. *et al.* On the dominance of filamentous cyanobacteria in shallow, turbid lakes. Ecology 78, 272–282 (1997).

[b11] Pfandl K. & Boenigk J. Stuck in the mud- suspended sediments as a key issue for survival of chrysomonad flagellates. Aquat. Microb. Ecol. 45, 89–99 (2006).

[b12] Cleven E. J. & Weisse T. Seasonal succession and taxon-specific bacterial grazing rates of heterotrophic nanoflagellates in Lake Constance. Aquat. Microb. Ecol. 23, 147–161 (2001).

[b13] Pernthaler J. Predation on prokaryotes in the water column and its ecological implications. Nat. Rev. Microbiol. 3, 1–10 (2005). 10.1038/nrmicro1180 15953930

[b14] Edmundson J. M. & Koenings J. P. The influence of suspended glacial particles on the macrozooplankton community structure within glacial lakes. Alaska Department of Fish and Game, Division of Fisheries Rehabilitation, Enhancement and Development, Nr. 67 (1986).

[b15] Chanudet V. & Filella M. Size and composition of inorganic colloids in a peri-alpine, glacial flour-rich lake. Geochim. Cosmochim. Acta 72, 1466–1479 (2008).

[b16] Caron D. A. Grazing of attached bacteria by heterotrophic microflagellates. Microb. Ecol. 13, 203–218 (1987). 24213296 10.1007/BF02024998

[b17] Boenigk J. & Arndt H. Comparative studies on the feeding behaviour of two heterotrophic nanoflagellates: filter-feeding choanoflagellate *Monosiga ovata* and the raptorial-feeding kinetoplastid *Rhynchomonas nasuta*. Aquat. Microb. Ecol. 22, 243–249 (2000).

[b18] Alldredge A. & Silver M. Characteristics, dynamics and significance of marine snow. Prog. Oceanogr. 20, 41–82 (1988).

[b19] Grossart H. P. & Simon M. Limnetic macroscopic organic aggregates (lake snow): occurrence, characteristic, and microbial dynamics in Lake Constance. Limnol. Oceanogr. 38, 532–546 (1993).

[b20] Seki H., Shortreed K. S. & Stockner J. G. Turnover rate of dissolved organic materials in glacially-oligotrophic and dystrophic lakes in British Columbia, Canada. Arch. Hydrobiol. 90, 210–216 (1980).

[b21] Cowan E. A. Characteristics of suspended particulate matter and sedimentation of organic carbon in Glacier Bay fjords. in Third Glacier Bay Science Symposium 1993 [Engstrom D. R. (ed.)], Glacier Bay National Park & Preserve, AK. U. S. National Park Service. 24–28 (1995).

[b22] Stibal M. & Šabacká Zarky J. Biological processes on glacier and ice sheet surfaces. Nat. Geosci. 5, 771–774 (2012).

[b23] Nesje A. & Dahl S. O. The Greenland 8200 cal. Yr. BP event detected in loss-on-ignition profiles in Norwegian lacustrine sediment sequences. J. Quarter. Sci. 16, 155–166 (2001).

[b24] Novarino G. A companion to the identification of cryptomonad flagellates (Cryptophyceae = Cryptomonadea). Hydrobiologia 502, 225–270 (2003).

[b25] Guillard R. R. L. Culture of phytoplankton for feeding marine invertebrates. in Culture of Marine Invertebrate Animals [Smith W. L., & Chanley M. H. (Eds.) 26–60 [Plenum Press, New York, USA (1975)].

[b26] Bonalumi M. *et al.* Particle dynamics in high-Alpine proglacial reservoirs modified by pumped-storage operation. Wat. Res. Res. 47, W09523, 10.1029/2010WR010262 (2011).

[b27] Massana R. & Güde H. Comparison between three methods for determining flagellate abundance in natural waters. Ophelia 33, 197–203 (1991).

[b28] Sherr E. B. & Sherr B. F. Protistan grazing via uptake of fluorescently labelled prey. in Handbook of methods in Aquatic Microbial Ecology [Kemp P. F., Sherr B. F., Sherr E. B., & Cole J. J. (eds.)] 695–701 [Lewis Publishers CRC Press LLC, Boca Raton (1993)].

[b29] Zar J. H. Biostatistical Analysis (2nd ed.). Prentice-Hall, Englewood Cliffs, N. J. (1984).

