# Supplementary material for: Negative consequences of glacial turbidity for the survival of freshwater planktonic heterotrophic flagellates
Source: Sci Rep. 2014 Feb 17;4:4113. doi: 10.1038/srep04113 (PMC3925964; doi:10.1038/srep04113)
Supplement: Supplementary Information — for Negative consequences of glacial turbidity for the survival of freshwater planktonic heterotrophic flagellates by Sommaruga and Kandolf [file srep04113-s1.pdf]

Supplementary Information Guide for

Negative consequences of glacial turbidity for the survival of freshwater planktonic heterotrophic flagellates

Ruben Sommaruga & Georg Kandolf
Correspondence to: ruben.sommaruga@uibk.ac.at

Supplementary Methods

Supplementary Figures S1, S2, S3, and S4

Supplementary Table S1

Supplementary Methods:

Characterization of glacial particles. For the mineralogical characterization, between 0.1–0.5 L of lake water (FAS 1 and FAS 3) was filtered through a glass fiber filter (Whatman GF/F) previously washed with Milli-Q water and burned in a muffle furnace at 450°C. For the crystalline phase analysis, the pulverized samples were examined by X-ray powder diffraction (Bruker-AXS D8) using the following setup: parallel beam optics, copper as target, 40 kV, 40 mA, E-dispersive counter, optical mode 2–70° theta/2Theta, 0.02° step-size, and 32 s counting time. The quantitative analysis of the phase content was done with Siroquant Analytical Software (Sietronics Pty Ltd.). The elemental composition (C/N) of glacial flour was determined using an Elemental Analyzer (Thermo–Flash EA 1112) running with the configuration for NC soils determination and calibrated with a four-point curve based on acetanilide. The weight loss on ignition (LOI) was also measured as a proxy for the organic coating in glacial 'flour'. The dried particles were weighed at room temperature and then the sediment was burned at 550°C for 2 h and weighed again. The LOI was calculated as the percentage of the dry weight. Because the ratio of particles to bacteria could be a decisive factor to explain the interference with HNF feeding, we measured the abundance of glacial 'flour' particles in a sample of 14 nephelometric turbidity units (NTU), i.e., the turbidity of Lake FAS 1 at the time of sample collection, with a 'Coulter Counter' (Coulter Multisizer II). The desired turbidity (see below) was obtained by suspending the dry particles in Milli-Q water at a concentration of 57 mg L^{-1} (14 NTU). Since the capillaries of the instrument are easily clogged with particle aggregates, the samples were treated in an ultrasonic bath for 20 s directly before measuring. Two capillaries with different size ranges were used to analyze the sample. One run was done with the capillary of 30 µm diameter (range 0.7–4 µm) to assess the lower range of the particle size spectrum. For this analysis, the sample was first filtered through a 5 µm pore size polycarbonate filter to exclude large particles and to avoid clogging of the capillary. Another run was done using the 100 µm diameter capillary (range

1.7–56.7 µm) to assess the upper size range of particles. The smallest capillary of 15 µm diameter was impossible to use due to clogging. Thus, the results are presented for particles between 0.7–56.7 µm. Though this method is not perfect because the presence the smallest fraction (< 0.7 µm) could not be resolved, it allows for estimating the abundance of glacial particles in the range that HNF prefer to graze on bacteria (i.e., 1-3 µm, Ref. 13). However, the total abundance of particles estimated by this method should be considered as underestimated. To have a better characterization of the size particle distribution, we used high-resolution particle size analysis by laser diffractometry[30] run in a Mastersize 2000 equipment coupled with a wet dispersion unit Hydro 2000MV (Malvern). The dry particles were dispersed in Milli-Q water and measured without or with ultrasound treatment (13.5 W for 1 and 3 min). This method gives information on scattered volume (%) for the different particle sizes. Finally, images of the glacial particles were made with a Phenom G2 scanning electron microscope at 8000 X magnification.

Reference

30. Sperazza, M., Moore, J. N. & Hendrix, M. S. High-resolution particle size analysis of naturally occurring very fine-grained sediment through laser diffractometry. *J. Sediment. Res.* **74**, 736-743 (2004).

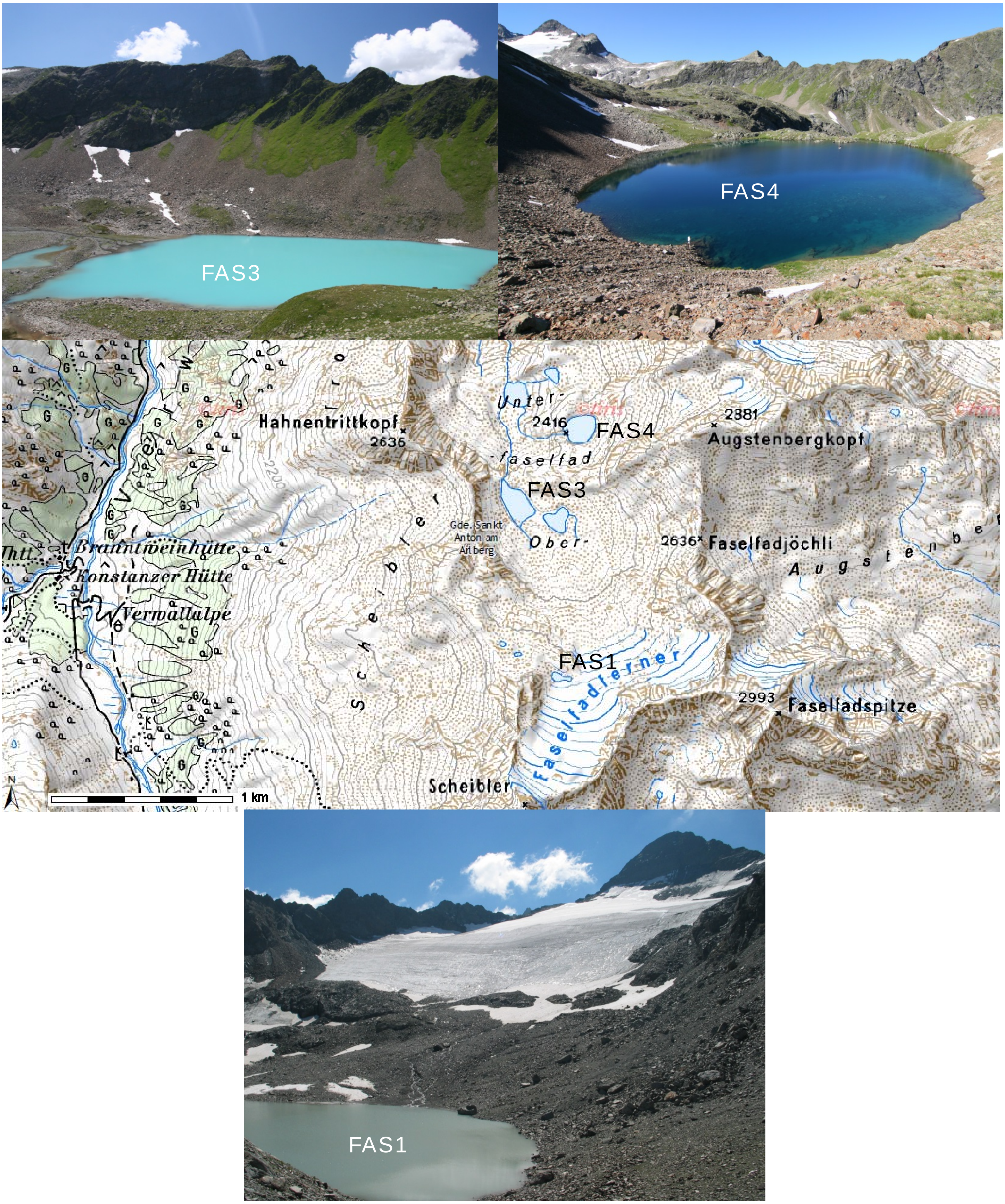

Supplementary Fig. S1. Lake FAS1 (2640 m a.s.l., max. depth: 4 m) is situated just below the glacier (Faselfadferner in the map) terminus, receives a large input of glacial ‘flour’, and consequently, it has the highest turbidity. Lake FAS3 (2420 m a.s.l., max. depth: 17 m) receives water through small proglacial streams, mainly flowing underground, probably from FAS1 and the glacier itself. Lake FAS4 is situated across a ridge at the same altitude as FAS3 (2400 m a.s.l., max depth 15 m), has no connection to the glacier anymore and is hence clear. Photo source: R. Sommaruga. Map source: Tiris, Tyrolean Government.

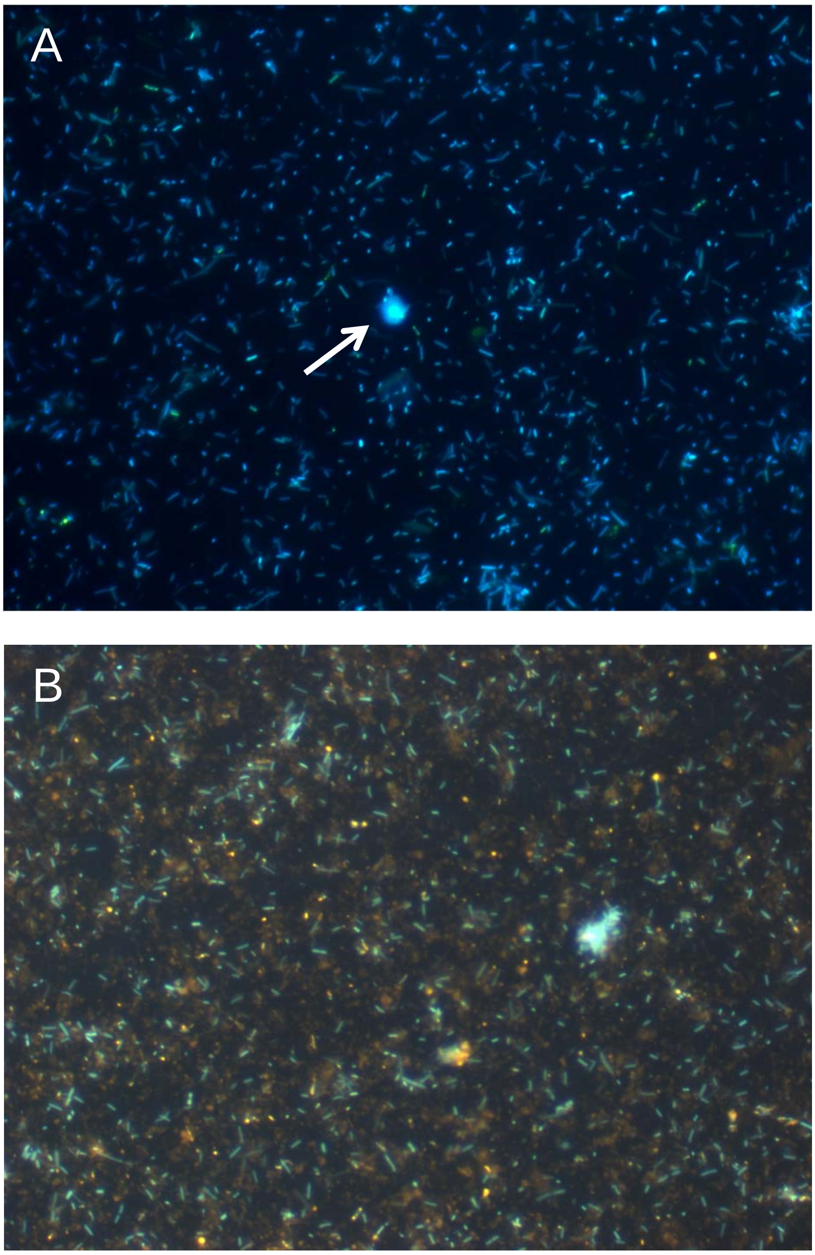

Supplementary Fig. S2. DAPI-stained bacteria in Lake FAS4 (A) and in Lake FAS3 (B). The arrow in A shows a heterotrophic nanoflagellate. Glacial ‘flour’ in B appears as yellow objects.

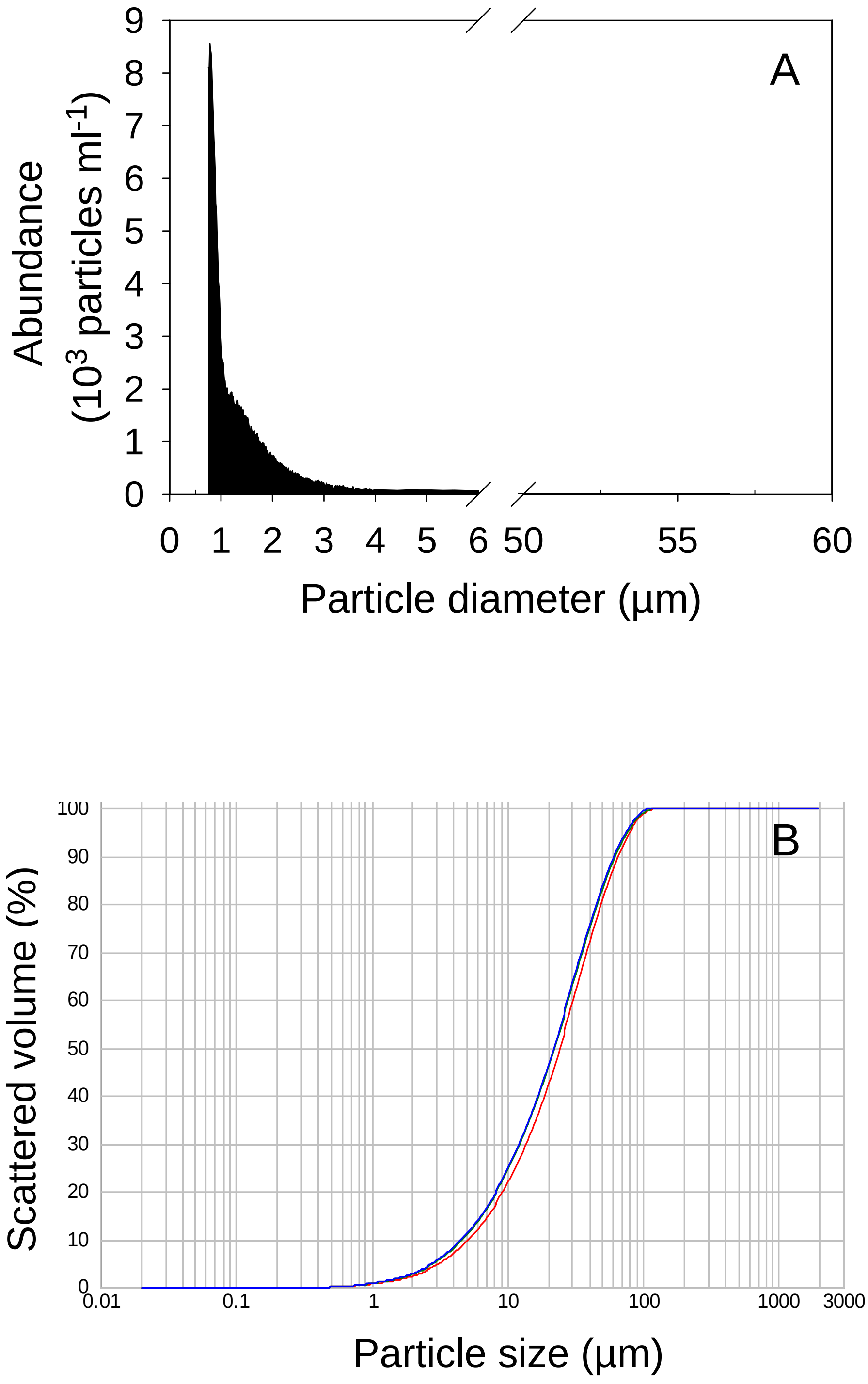

Supplementary Fig. S3. Abundance of glacial particles at 14 NTU as measured with a Coulter Counter (A) and scattered volume distribution (%) for different size particles measured by laser diffractometry (B). The red line represents measurements done without sonication and the blue one after 3 min sonication.

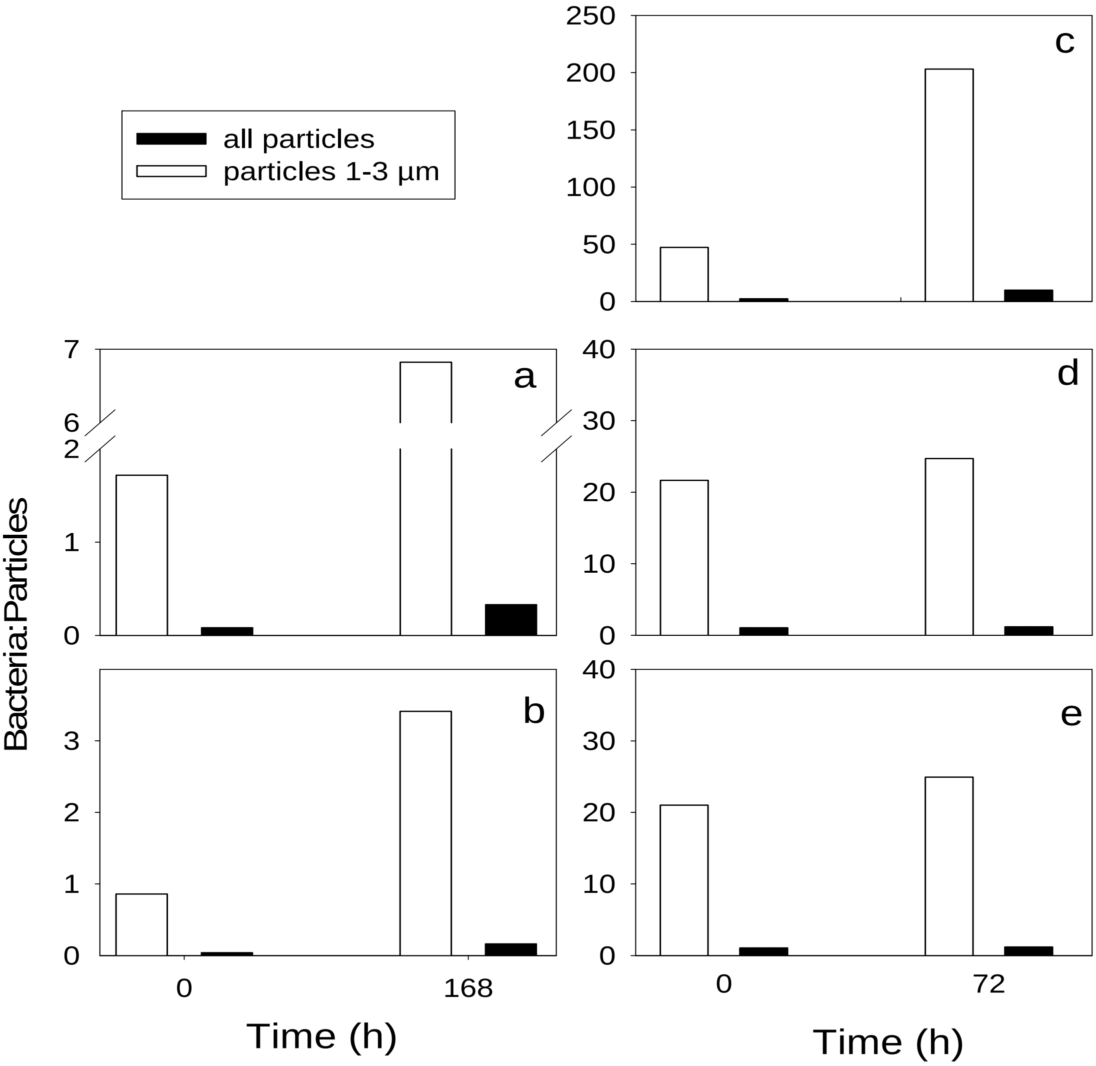

Supplementary Fig. S4. Bacteria:particle ratio in the 14 NTU (a) and 30 NTU (b) treatment during the experiment with the natural HNF community from Lake FAS4 and in the *Spumella* sp. (c, shown only for the 14 NTU treatment) and *Dinobryon divergens* experiments done in the dark (d) or in the presence of light (e). 'All particles' represents the total particle abundance measured with the 'Coulter Counter'.

Supplementary Table S1. Water temperature, turbidity, and abundance of bacteria (DAPI-positive), heterotrophic (HNF), and autotrophic (ANF) nanoflagellates in Faselfad lakes 1, 3, and 4, on 8 August 2009.

Lake	Sampling depth (m)	Temperature (°C)	Bacterial abundance (10^5 cells ml^{-1})	HNF abundance (10^3 cells ml^{-1})	ANF abundance (10^4 cells ml^{-1})
FAS1	1	6.1	1.93	-	0.04
FAS3	1	9.5	2.99	-	0.71
	6	7.1	4.45	-	1.79
	10	6.4	5.12	-	2.04
	15	5.3	5.29	-	1.43
FAS4	1	11.0	4.14	0.85	0.16
	6	9.6	5.12	2.25	0.27
	10	6.9	8.32	2.57	0.31
	15	7.3	8.67	2.44	0.33
